# The mitochondrial and plastid genomes of *Volvox carteri*: bloated molecules rich in repetitive DNA

**DOI:** 10.1186/1471-2164-10-132

**Published:** 2009-03-26

**Authors:** David Roy Smith, Robert W Lee

**Affiliations:** 1 Department of Biology, Dalhousie University, Halifax, Nova Scotia, Canada

## Abstract

**Background:**

The magnitude of noncoding DNA in organelle genomes can vary significantly; it is argued that much of this variation is attributable to the dissemination of selfish DNA. The results of a previous study indicate that the mitochondrial DNA (mtDNA) of the green alga *Volvox carteri *abounds with palindromic repeats, which appear to be selfish elements. We became interested in the evolution and distribution of these repeats when, during a cursory exploration of the *V. carteri *nuclear DNA (nucDNA) and plastid DNA (ptDNA) sequences, we found palindromic repeats with similar structural features to those of the mtDNA. Upon this discovery, we decided to investigate the diversity and evolutionary implications of these palindromic elements by sequencing and characterizing large portions of mtDNA and ptDNA and then comparing these data to the *V. carteri *draft nuclear genome sequence.

**Results:**

We sequenced 30 and 420 kilobases (kb) of the mitochondrial and plastid genomes of *V. carteri*, respectively – resulting in partial assemblies of these genomes. The mitochondrial genome is the most bloated green-algal mtDNA observed to date: ~61% of the sequence is noncoding, most of which is comprised of short palindromic repeats spread throughout the intergenic and intronic regions. The plastid genome is the largest (>420 kb) and most expanded (>80% noncoding) ptDNA sequence yet discovered, with a myriad of palindromic repeats in the noncoding regions, which have a similar size and secondary structure to those of the mtDNA. We found that 15 kb (~0.01%) of the nuclear genome are homologous to the palindromic elements of the mtDNA, and 50 kb (~0.05%) are homologous to those of the ptDNA.

**Conclusion:**

Selfish elements in the form of short palindromic repeats have propagated in the *V. carteri *mtDNA and ptDNA, resulting in the distension of these genomes. Copies of these same repeats are also found in a small fraction of the nucDNA, but appear to be inert in this compartment. We conclude that the palindromic repeats in *V. carteri *represent a single class of selfish DNA and speculate that the derivation of this element involved the lateral gene transfer of an organelle intron that first appeared in the mitochondrial genome, spreading to the ptDNA through mitochondrion-to-plastid DNA migrations, and eventually arrived in the nucDNA through organelle-to-nucleus DNA transfer events. The overall implications of palindromic repeats on the evolution of chlorophyte organelle genomes are discussed.

## Background

The amount of noncoding DNA (i.e., intronic and intergenic DNA) in organelle genomes varies significantly. One evolutionary lineage where the gamut of organelle-genome compactness is particularly pronounced is the Chlorophyta (a phylum containing most known classes of green algae [1]) for which the noncoding-DNA contents span from 10% (*Ostreococcus tauri*) to 53% (*Pseudendoclonium akinetum*) for mitochondrial DNA (mtDNA) and from 5% (*Helicosporidium *sp. ex *Simulium jonesii*) to 56% (*Chlamydomonas reinhardtii*) for plastid DNA (ptDNA). Although the processes influencing genome compactness are poorly understood, it is suggested that they are associated with the proliferation of selfish DNA [2-4], which we will define for the purpose of this study as any noncoding DNA element with the ability to spread its sequence to new genomic locations. Selfish DNA is ubiquitous in eukaryotic nuclear genomes and is also present, though less pervasive, in organelle genomes [5-7], including those of chlorophytes [8] – and see Hurst and Werren [9] for a review.

One proposed type of selfish DNA that is often observed in the mitochondrial and plastid genomes of chlorophytes are short palindromic repeats; these repetitive elements are marked by their short length [10–100 nucleotides (nt)] and their ability to be folded into hairpin (i.e., stem-loop) structures. Short palindromic repeats have been identified in the mtDNA and ptDNA from a variety of chlorophyte taxa (for compilations see [6,10-13]), and there are indications that the organelle genomes of some chlorophyte species are more prone to the dissemination of palindromic repeats than those of other chlorophyte species. For example, the mtDNA of *P. akinetum*, the largest chlorophyte mitochondrial genome sequenced to date [95 kilobases (kb)], contains a series of short palindromic repeats that have proliferated throughout its intergenic and intronic regions [11]; moreover, these same palindromic elements are found in the noncoding regions of the *P. akinetum *ptDNA [12], indicating that the lateral transfer of repetitive DNA between organelle compartments is taking place – it is speculated that this process involves the hitchhiking of repeats on introns that migrate between the mtDNA and ptDNA [12]. Short palindromic repeats in chlorophyte organelle DNA, as well as adding to the amount of noncoding DNA in a genome, have been credited for: 1) genome rearrangements [10], including the fragmentation and scrambling of ribosomal-RNA (rRNA) coding modules [14]; 2) changes in genome conformation and chromosome number [13,15]; and 3) having regulatory functions [13,16].

A previous study found that the mitochondrial genome of the multicellular, chlorophyte green alga *Volvox carteri*, a close relative to *C. reinhardtii*, is profuse with short palindromic repeats [17]. By sequencing ~8 kb of mtDNA, corresponding to portions of *cob*, *cox1*, and their group-I introns, and an intergenic region between *nad2 *and *nad6*, these authors showed that at least two group-I introns and one intergenic region of the *V. carteri *mitochondrial genome contain extensive palindromic sequences. Ten classes of short palindromic repeats, all of which share sequence identity with one another, were discerned. Aono et al. [17] concluded that these repetitive elements are selfish DNA, that they are expanding the intronic and intergenic regions of the mitochondrial genome, and that they may have played a role in the fragmentation of rRNA-coding modules.

In June 2007, the United States Department of Energy Joint Genome Institute (DOE JGI) released the draft nuclear genome sequence of *V. carteri *[18]. This genome is ~140 megabases (Mb) in length [18-20], which is the largest chlorophyte nuclear genome sequence currently available (see [21] for a compilation), and the preliminary annotation of this sequence suggests that it is rich in noncoding DNA, much of which appears to be selfish [18].

Our interest regarding the evolution of selfish DNA in *V. carteri *was sparked when we located short palindromic repeats in the *V. carteri *draft nuclear genome sequence that are similar to those in the mtDNA. To see if palindromic repeats are also present in the *V. carteri *ptDNA, we PCR amplified and sequenced an intergenic region from the plastid genome; our analysis of this sequence confirmed that the ptDNA contains palindromic repeats that have a similar size and potential secondary structure to those in the mitochondrial compartment. We, therefore, set out to do a detailed investigation of the palindromic repeats in *V. carteri *by sequencing and characterizing large portions of mtDNA and ptDNA and then comparing these data to the nuclear DNA (nucDNA). Our motives for this study are to gain insights into the role selfish DNA plays in organelle-genome expansion and its ability to move between genetic compartments.

## Results

### General features of the mitochondrial- and plastid-DNA sequences from *V. carteri*

**Figure 1:**
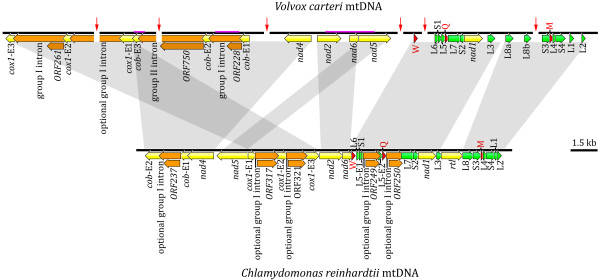
**Partial genetic map of the *Volvox carteri *mitochondrial genome compared to the complete mtDNA genetic map of *Chlamydomonas reinhardtii***. Protein-coding regions are yellow and their exons are labelled with an "E" followed by a number denoting their position within the gene. Introns and their associated open reading frames are orange. Transfer RNA-coding regions are red; they are designated by the single-letter abbreviation of the amino acid they specify. The large-subunit and small-subunit rRNA-coding modules are green. Arrows within the coding regions denote their transcriptional polarities. Solid red arrows perpendicular to the genome map indicate regions of the genome assembly where sequence data is either unreadable or lacking. The mtDNA regions that were previously sequenced and described by Aono et al. [17] are underlined in pink on the genome map. Gray blocks highlight regions of synteny between the *V. carteri *and *C. reinhardtii *mitochondrial genomes. Note: the optional group-I intron in *cox1 *is found in the mtDNA of *V. carteri *strain HK10 (UTEX 1885); this intron is absent from *V. carteri *strain 72-52 (UTEX 2908) – the *C. reinhardtii *strains in which the different introns occur are listed in [50].

**Figure 2:**
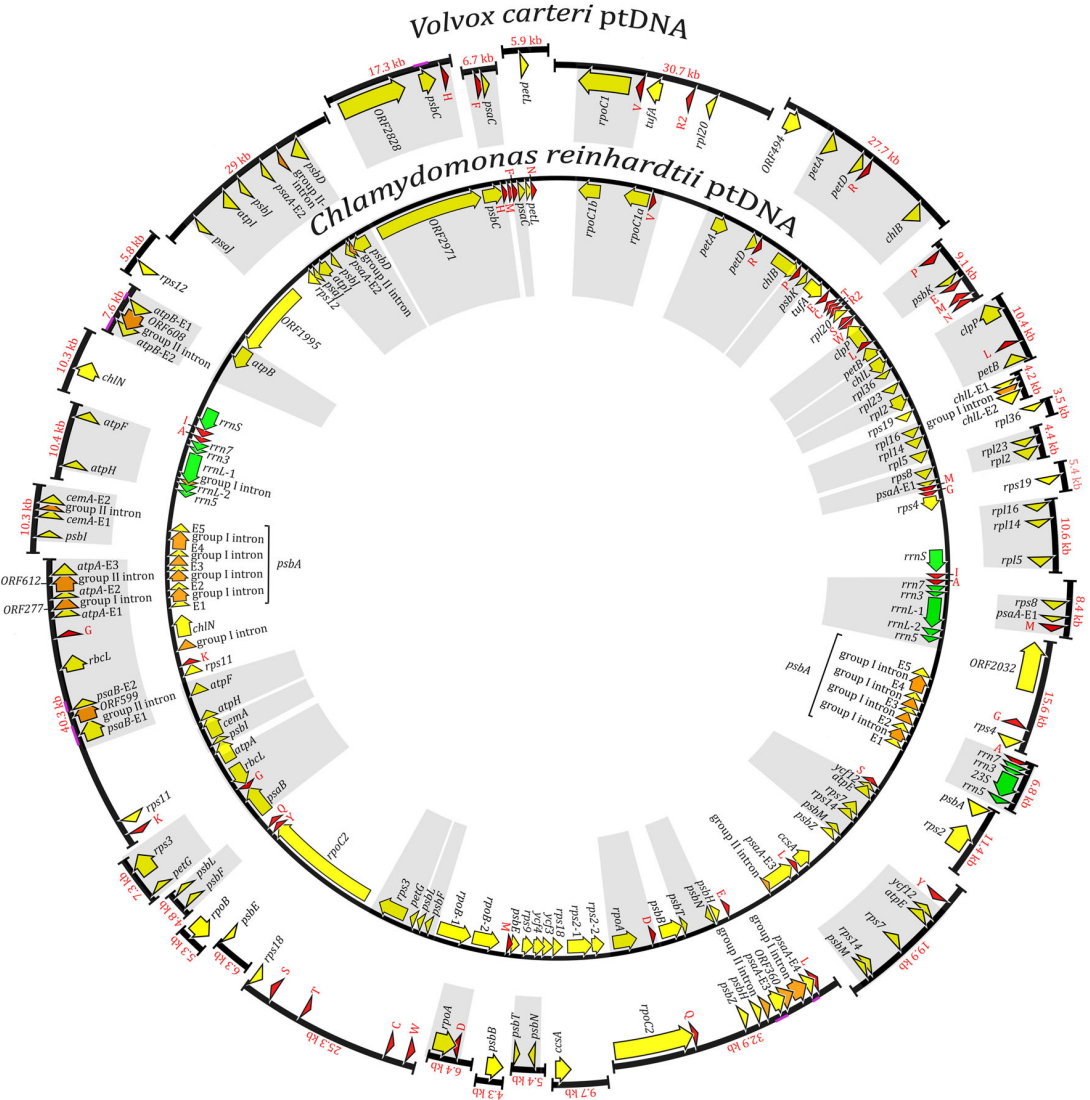
**Partial genetic map of the *Volvox carteri *plastid genome compared to the complete ptDNA genetic map of *Chlamydomonas reinhardtii***. Regions encoding proteins are yellow and their exons are labelled with an "E" followed by a number signifying their order within the gene. Introns and their associated open reading frames are orange. Transfer RNA-coding regions are red; they are designated by the single-letter abbreviation of the amino acid they specify. Ribosomal RNA-coding regions are green. The coding regions are shaped into arrows that denote their transcriptional polarities. Regions previously described by Aono et al. [17] are underlined in pink. Gray blocks highlight regions of synteny between the *V. carteri *and *C. reinhardtii *plastid genomes. Note, the true order of the *V. carteri *contigs are unknown.

Using a long-range PCR approach in conjunction with cloning, we sequenced from *V. carteri *29,961 nt of the mitochondrial genome and 420,650 nt of the plastid genome; partial genetic maps of these genomes describing the coding and noncoding regions that were sequenced are respectively shown in Figures 1 and 2. Regions of the *V. carteri *organelle DNA that were previously characterized (~8 kb of mtDNA and ~5 kb of ptDNA) are highlighted in pink on these maps. Although we attempted to completely sequence the mitochondrial and plastid genomes, presumed secondary structures in the mtDNA and ptDNA templates likely caused many of the sequencing reactions to suddenly stop – even when using protocols designed to alleviate this problem [22]. Furthermore, the repetitive nature of the organelle genomes means that much of the mtDNA and ptDNA sequence data are irresolvable using the currently available genome-assembly software programs: many of the organelle intergenic and intronic DNA sequences collapse into networks of spurious repetitive motifs upon assembly. Moreover, the fact that most of the mtDNA and ptDNA intergenic regions are much longer than a typical sequencing read (some intergenic regions exceed 15 kb) means that these collapsed repeats are irresolvable. At present, the most sophisticated assembly programs use the paired-end sequencing data from whole-genome shotgun reads to resolve complex repeat regions. Because there is a *V. carteri *nuclear genome sequencing project [18], we have access to paired-end sequencing reads for the mitochondrial and plastid genomes (see Methods for details); but even with these data, neither the assembly programs nor our own manual, by-eye assembly methods can untangle these repeats. Because of these difficulties, our *V. carteri *mitochondrial-genome assembly, although contained in a single contig, contains six regions where the mtDNA sequence is either unreadable or unavailable (Figure 1), and the assembly of the ptDNA is divided into 34 contigs (Figure 2). Nevertheless, we did sequence and characterize enough mtDNA and ptDNA to confidently describe the abundance and various types of noncoding DNA in each of these organelle genomes.

The organelle-DNA sequences presented in this study were validated by collecting and assembling mtDNA and ptDNA sequence data that were generated by the DOE JGI *V. carteri *nuclear genome sequencing project [18]. This was performed by: 1) downloading DNA-sequence trace files corresponding to the *V. carteri *mitochondrial and plastid genomes; 2) assembling these trace files into contigs; and 3) mapping the trace-file contigs to the *V. carteri *mtDNA and ptDNA sequences produced in this study. Ultimately, the mtDNA and ptDNA sequences coming from the DOE JGI covered all of our self-generated *V. carteri *sequence data with >50-fold redundancy. It is important to note that the DOE JGI data that we used to confirm our mtDNA and ptDNA sequences came from *V. carteri *strain HK10 (UTEX 1885), whereas the *V. carteri *mtDNA and ptDNA sequences that we generated came from strain 72-52 (UTEX 2908), which is a dissociator mutant derived from HK10 [23,24]. In all instances, the organelle DNA sequence data coming from strain HK10 were identical to those of strain 72-52 (i.e., no ambiguities between the DOE JGI trace-file contigs and our sequences were observed), with the exception of a group-I intron that is present in the mtDNA of HK10 but absent in that of 72-52 (see below for details).

**Figure 3:**
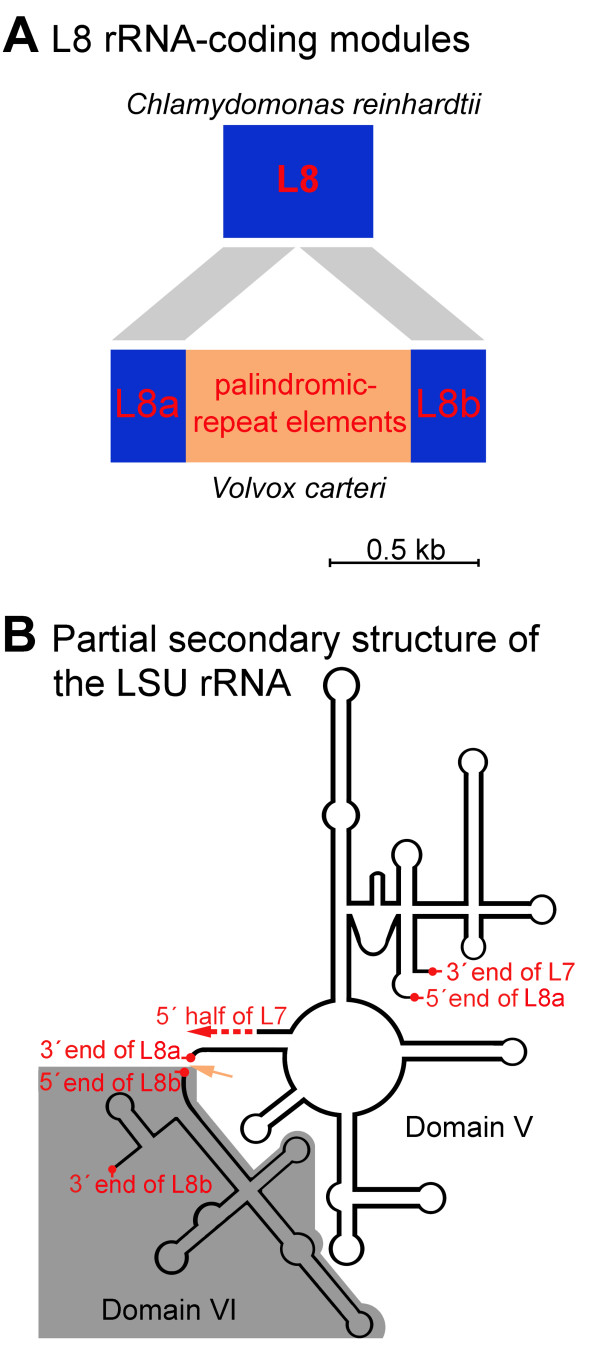
**Schema of the L8 rRNA-coding module in the *Volvox carteri *mitochondrial genome and its relationship to that in the *Chlamydomonas reinhardtii *mtDNA**. The grey bars in **A **denote regions of sequence identity between the L8 rRNA-coding modules of the *V. carteri *and *C. reinhardtii *mtDNA. **B **depicts the *V. carteri *L8 coding module in the context of the large-subunit (LSU) rRNA secondary-structure model; the orange arrow points to the repetitive region separating the L8a and L8b components of the L8 module. The LSU rRNA secondary-structure model is based on that of Boer and Gray [49].

Of the 29,961 nt of *V. carteri *mtDNA sequence data presented here, 18,355 nt (61%) are noncoding, which include 7,870 nt (26%) of intronic DNA and 10,485 nt (35%) of intergenic DNA; the remaining 11,606 nt (39%) are comprised of 8,166 nt (27%) coding for proteins and 3,440 nt (12%) coding for structural RNAs. The intergenic regions range from 0 to >1,400 nt in length, and, on average, are 455 nt long. The AT content of the 29,961 nt mtDNA sequence is 66%. Our annotation of the *V. carteri *mtDNA includes 7 protein-coding genes; the full suite of rRNA-coding modules required for the formation of the large-subunit and small-subunit rRNAs; 3 tRNA-coding genes; and 3 introns, 2 of group-I affiliation, located in *cox1 *and *cob*, and 1 of group-II affiliation, located in *cob *(Figure 1). Both group-I introns contain an open reading frame (ORF) encoding a putative LAGLIDADG endonuclease. The sole group-II intron has an ORF for which the deduced amino-acid sequence shows similarity to a reverse transcriptase (Figure 1). The DOE JGI *V. carteri *mtDNA sequences that we assembled (derived from *V. carteri *strain HK10) have, as mentioned above, an additional group-I intron in *cox1 *that is not present in *V. carteri *strain 72-52 (Figure 1). The coding suite that we acquired for the *V. carteri *mtDNA is identical to that of the *C. reinhardtii *mitochondrial genome [16,25,26] as is the gene order save for two rearrangements, which are outlined on Figure 1. There are two interesting features of the *V. carteri *mtDNA relative to its *C. reinhardtii *counterpart. First, the *V. carteri *L8 rRNA-coding module harbours a 725 nt insertion composed of short palindromic repeats, whereas that of *C. reinhardtii *contains no repeats (Figure 3a). When the *V. carteri *L8 module is folded into a putative secondary-structure model within the context of the LSU rRNA it contains two structural constituents: L8a and L8b (corresponding to the LSU rRNA domains V and VI, respectively), where the 3' end of L8a and the 5' end of L8b border the 725 nt insertion (Figure 3b). At present, we do not know if this insertion is removed from the primary transcript so that separate L8a and L8b mature transcripts are produced or if a single mature L8 transcript is generated with the insertion. The second point of interest is that although a putative reverse transcriptase gene is found in the mtDNA of both of *V. carteri *(*ORF750*) and *C. reinhardtii *(*rtl*), that of *V. carteri *appears to be part of a group-II intron located in *cob*, whereas in *C. reinhardtii*, *rtl *is a free standing gene that is lacking an intron but speculated to have originated from one [27,28] – see Popescu and Lee [29] for further discussion. The deduced amino-acid sequences of both *ORF750 *and *rtl *have a conserved domain that resembles that of a reverse transcriptase with group-II intron affiliation; however, the amino-acid sequence of *ORF750 *also has a conserved domain with similarity to a type-II intron maturase, while that of *rtl *does not. This extra domain encoded in *ORF750 *also explains why this ORF is twice the size of *rtl *(2,250 nt versus 1,119 nt).

In regard to the 420,650 nt of *V. carteri *ptDNA sequence data that were generated, 338,557 nt (80%) are noncoding, of which 16,005 nt are intronic DNA and 322,552 nt are intergenic DNA; 77,335 nt (19%) code for proteins and 4,758 nt (1%) code for structural RNAs. The intergenic regions that were sequenced range from 87 nt to >12,444 nt in length and have an average size of 5,103 nt. The 420 kb of ptDNA are 57% AT. Our annotation of the ptDNA sequences includes 91 genes: 60 coding for standard plastid proteins, 27 coding for structural RNAs (23 tRNAs and 4 rRNAs), and 4 corresponding to ORFs (*ORF494, ORF2032, ycf12, ORF2828*) that have been previously found in plastid genomes (Figure 2). Three group-I introns were observed, located in *chlL, psaA*, and *atpA*; those in the later two genes contain an ORF encoding a putative LAGLIDAD endonuclease. Five group-II introns were discerned, situated in *psaA*, *cemA, psaB*, *atpA*, and *atpB*; the introns of the latter two genes have an ORF for which the inferred amino-acid sequence resembles that of a reverse transcriptase. The group-II intron of *psaA *is fragmented into two separate modules, which is also the case for *C. reinhardtii *(Figure 2). The 91 *V. carteri *ptDNA genes presented here are all found in the *C. reinhardtii *plastid genome with the exception of *ORF494*. The only apparent homolog of *ORF494 *is the ribosomal operon-associated gene (*roaA*) found in the *Euglena gracilis *plastid genome. Note, the *C. reinhardtii *ptDNA encodes a further 4 tRNA-coding and one rRNA-coding regions that we were unable to amplify from *V. carteri*.

**Figure 4:**
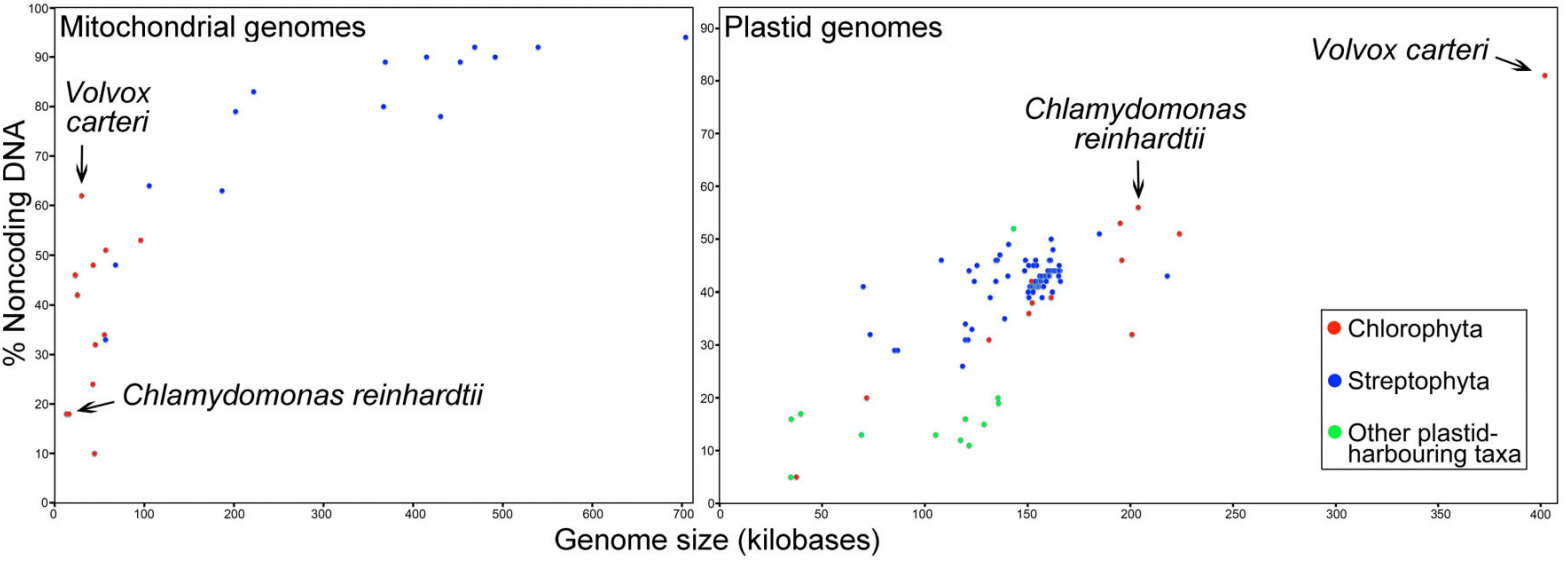
**Fraction of noncoding DNA plotted against genome size for the available organelle genomes from streptophytes, chlorophytes, and other plastid-harbouring taxa**. The data points corresponding to the mtDNA and the ptDNA of *V. carteri *and those of its close relative *C. reinhardtii *are labelled and marked with arrows on the appropriate graph. The noncoding-DNA contents and genome sizes from which these two graphs were plotted are listed in Supplementary Table S1 [see Additional file 1]. Values of 30 kb and 420 kb, respectively, were chosen, based on our sequence data, as minimum-size-estimates of the mitochondrial and plastid genomes.

A graph comparing both the estimated sizes and the fraction of noncoding nucleotides in the mitochondrial and plastid genomes of *V. carteri *relative to those of the currently available complete organelle-genome sequences from chlorophyte-, streptophyte- and other plastid-harbouring-taxa is shown in Figure 4 [and see Additional file 1]. Values of 30 kb and 420 kb, respectively, were chosen, based on our sequence data, as minimum-estimate genome sizes for the *V. carteri *mtDNA and ptDNA.

### Short palindromic repeats in the mitochondrial and plastid genomes of *V. carteri*

**Figure 5:**
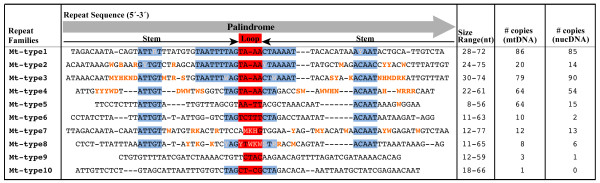
**Abundance and classification of the *Volvox carteri *mitochondrial-DNA palindromic repeat elements**. Regions of high sequence identity among the different repeat families are shaded in blue; variable sites are orange; the loop portions of the putative hairpin structures are shaded in red, and the stems (i.e., complementary bases) of these structures are located beneath the black arrows. Nuclear DNA analyses were performed using the first 75 scaffolds of the *V. carteri *draft nuclear genome sequence (version 1) at the DOE JGI [18].

**Figure 6:**
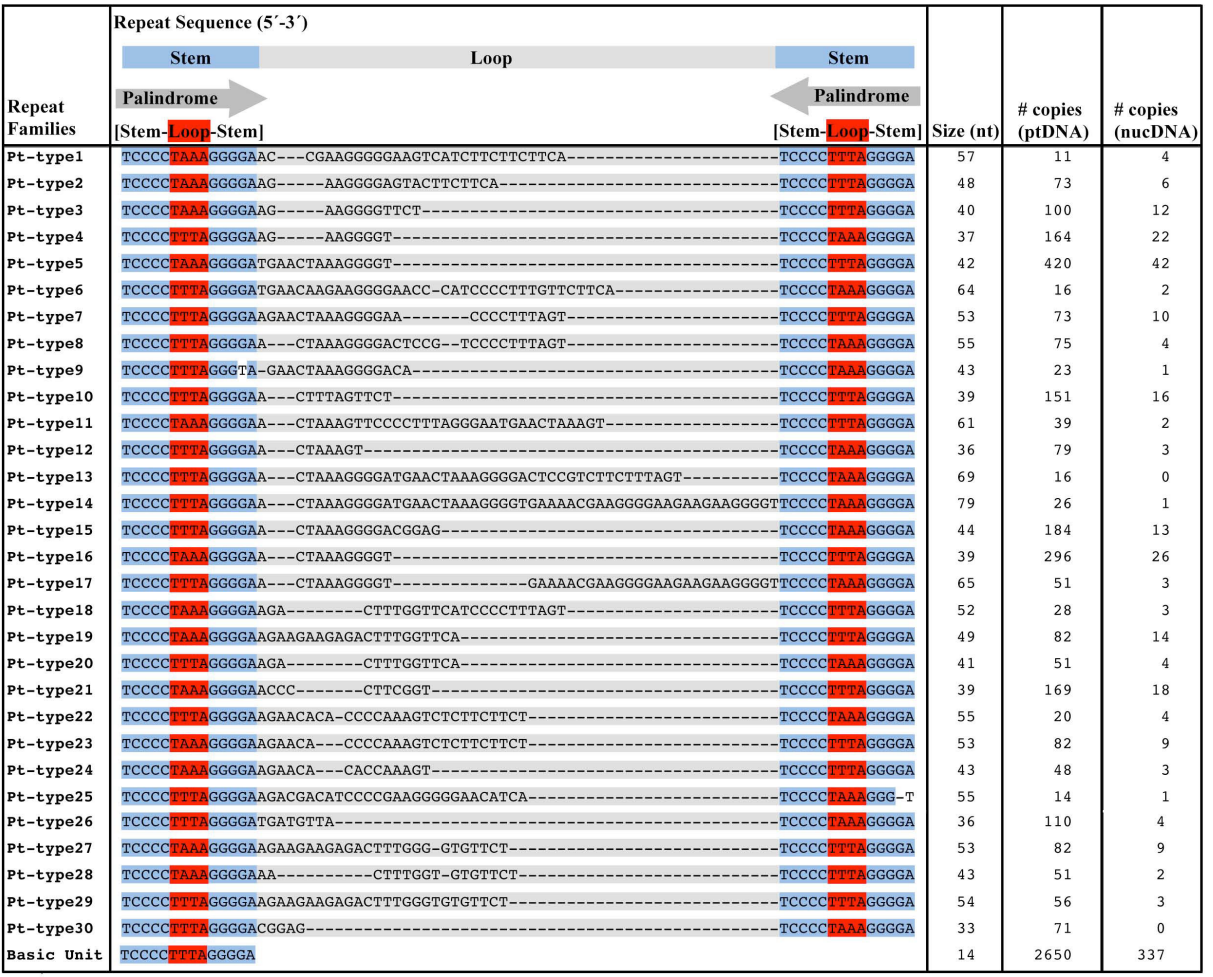
**Abundance and classification of the *Volvox carteri *plastid-DNA palindromic repeat elements**. Regions of high sequence identity among the different repeat families are shaded in blue; the loop portions of the putative hairpin structures are shaded in either red or grey, and the stems (i.e., complementary bases) of these structures are located beneath the grey arrows. Nuclear DNA analyses were performed using the first 75 scaffolds of the *V. carteri *draft nuclear genome sequence (version 1) at the DOE JGI [18].

Scanning of the *V. carteri *mitochondrial- and plastid-DNA sequences for repetitive elements lead to the identification of a series of short palindromic repeats in both of the organelle genomes; the consensus sequences, complementary bases, and copy numbers of the mtDNA and ptDNA palindromic elements are outlined in Figures 5 and 6, respectively. Although the short palindromic repeats of the mtDNA share many of the same structural traits as those of the ptDNA (discussed below), they differ by >50% in sequence identity and, therefore, must be considered as distinct repeats relative to those of the plastid genome.

The short palindromic repeats in the *V. carteri *mtDNA are restricted to intergenic and intronic regions, with the exception of the palindromic elements in the L8 rRNA-coding module. All of the intergenic regions that measure >50 nt in length consist predominantly of palindromic repeats; the few intergenic regions with lengths <50 nt are composed of non-repetitive DNA. Within the intronic regions, the palindromic repeats are confined to the non-ORF portions of the group-I and group-II introns. All four of the identified mitochondrial introns contain short palindromic repeats in their non-ORF regions, including the optional group-I intron of *cox1*, which was found in the mtDNA of *V. carteri *strain HK10. Approximately 14,600 nt (~80%) of the 18,355 nt of noncoding mtDNA that were sequenced are composed of short palindromic repeats. A dotplot similarity matrix of the *V. carteri *mtDNA plotted against itself, shown in Supplementary Figure S1 [see Additional file 2], emphasizes the magnitude of repetitive DNA in this genome and draws attention to the high degree of sequence identity between the different palindromic elements within and among the various intergenic and intronic regions.

The short palindromic repeat elements identified in the *V. carteri *mtDNA show >50% sequence identity with one another and share similar structural and compositional traits (Figure 5). The individual palindromes range from 11–77 nt in length (the average size is 50 nt) and from 71–84% in their AT content. When the palindromes are folded into hairpin structures, the stem component of the hairpin varies from 4–37 nt in length, and the loop portion is usually 3–5 nt long and frequently has the sequence 5'-TAAA-3' or 5'-TTTA-3' (Figure 5). In many instances, a short palindromic repeat is found inserted within another palindromic repeat, resulting in larger, more elaborate repetitive elements; these larger repeats have a maximum length of 633 nt, and, in a few cases, are found at multiple locations in the mitochondrial genome. For example, a 550 nt repeat sequence composed of complete and incomplete short palindromic units is found in the group-I introns of *cob *and *cox1*, in the intergenic regions between *cob *and *nad4*, and in the group-II intron of *cob*. Some of these more complex repeats can also be folded into tRNA-like structures – as shown in Supplementary Figure S2 [see Additional file 3].

Like the mitochondrial genome, the noncoding regions of the *V. carteri *plastid genome abound with short palindromic sequences. These palindromic elements are observed in all of the sequenced intergenic regions that have lengths >100 nt and in the non-ORF portions of the *psaA *and *atpA *group-I introns. No palindromic elements are located in the *chlL *group-I intron or in any of the identified group-II introns. Overall, the short palindromic repeats constitute ~80% (~270 kb) of the 338,557 noncoding nucleotides in the *V. carteri *ptDNA. A dotplot similarity matrix of the *V. carteri *plastid genome plotted against itself (Supplementary Figure S3 [see Additional file 4]) shows the high level of sequence identity among the various palindromic repeats.

In the *V. carteri *ptDNA, most of the short palindromic repeats contain the sequence motif 5'-TCCCCTTTAGGGA-3' (Figure 6). The palindromes have a size range of 14–79 nt, with an average length of 50 nt, and, when folded into hairpin structures, their stems and loops vary in length from 5–29 nt and 3–5 nt, respectively. In most cases, the loops of the hairpin structures contain the sequence 5'-TAAA-3' or 5'-TTTA-3' (Figure 6). The AT content of the ptDNA palindromes varies from 39–55%. As observed for the mtDNA, the ptDNA palindromic elements are often found inserted into one another, the consequence of which is a series of multifarious repetitive sequences.

The short palindromic repeats of the mitochondrial and plastid compartments have similar structural attributes: 1) they have proliferated in intergenic regions and non-ORF segments of introns; 2) they have an average size of 50 nt and a maximum length of ~78 nt; and 3) the loops of their hairpin structures are generally 3–5 nt long with the sequence 5'-TTTA-3' or 5'-TTTA-3'.

### The nuclear genome of *V. carteri *shares sequence identity with organelle DNA

To investigate if the short palindromic repeats in the mtDNA and ptDNA of *V. carteri *are present in the nucDNA, we analyzed the draft nuclear genome sequence of *V. carteri *at the DOE JGI [18]. Manual curation of the *V. carteri *nuclear genome is still underway; therefore, only the first 75 scaffolds of the nuclear-genome assembly were analyzed. Approximately 78% of the *V. carteri *nucDNA is contained in these 75 scaffolds, their cumulative length is 109.2 Mb, and each scaffold is at least 0.5 Mb long. The amount of nucDNA in these 75 scaffolds that map to mtDNA and ptDNA is described in Table 1; the approximate number of nucDNA-located organelle-like repeats is outlined in Figures 5 and 6.

**Table 1:** Amount of nuclear DNA in *Volvox carteri *that maps to the mitochondrial and plastid genomes.

		**# of similarity regions^a^**	**Avg. length of similarity region (nt)**	**Max similarity length (nt)**	**Cumulative lengths of similarity regions (nt)**	**Fraction of nuclear genome**
Amount of nucDNA mapping to the mitochondrial genome (by mtDNA subcategory^b^)	Protein-coding genes^c^	114	64	291	7335	6.70 × 10^-5^
	
	Sructural-RNA genes^d^	73	40	207	2969	2.70 × 10^-5^
	
	Intronic ORFs^e^	278	30	233	278	0.25 × 10^-5^
	
	Intergenic and non-ORF intronic regions^f^	337	38	933	14782	13.53 × 10^-5^
	
	Subtotal	802	39	933	33452	30.63 × 10^-5^

Amount of nucDNA mapping to the plastid genome (by ptDNA subcategory^b^)	Protein-coding genes^c^	365	48	462	17440	15.90 × 10^-5^
	
	Structural-RNA genes^d^	127	31	170	4008	3.60 × 10^-5^
	
	Intronic ORFs^e^	31	34	154	1075	0.98 × 10^-5^
	
	Intergenic and non-ORF intronic regions^f^	927	22	3430	50631	46.36 × 10^-5^
	
	Subtotal	1450	29	3430	73154	66.99 × 10^-5^

Total nucDNA mapping to organelle DNA		2252	33	3430	106606	97.62 × 10^-5^

Note: Nuclear DNA analyses are based on the *V. carteri *draft nuclear genome sequence (version 1) at the DOE JGI [18]. Only the first 75 scaffolds of the nuclear-genome assembly were analyzed; approximately 78% of the *V. carteri *nucDNA is contained in these 75 scaffolds and their cumulative length is 109.2 Mb.

^a ^The number of distinct regions in the *V. carteri *nucDNA that show >90% sequence identity and at least 25 nt of aligned length to organelle DNA.

^b ^Refers to the region of the organelle genome to which the nucDNA maps.

^c ^Includes all of the identified protein-coding genes.

^d ^Includes all of the identified tRNA- and rRNA-coding genes.

^e ^Includes all of the identified group I and II intronic-ORFs.

^f ^Includes all of the identified intergenic and non-ORF intronic regions.

Thirty-three kilobases of nucDNA (~0.03% of the nuclear genome) share >90% identity with mtDNA; 14.7 kb (44%) of this shared sequence are homologous to the short palindromic repeats in the intergenic and non-ORF intronic regions of the mitochondrial genome; the remaining 10.6 kb (56%) map to the coding and intronic-ORF portions of the mtDNA (Table 1). In the nucDNA, 802 distinct regions show homology to mtDNA; the average mapping length of these regions is 39 nt. Of the 75 nuclear scaffolds that were analyzed, all but two (scaffold 66 and 75) have at least one region that shows homology to mtDNA.

Seventy-three kilobases of the *V. carteri *nucDNA (~0.07% of the nuclear genome) share >90% identity with ptDNA; 50.6 kb (69%) of this shared sequence are homologous to the short palindromic repeats in the intergenic and non-ORF-intronic portions of the ptDNA, and 22.5 kb (31%) are homologous to the coding regions and intronic ORFs of the plastid genome (Table 1). In the nuclear genome, 1,450 different regions show homology to ptDNA, and the average similarity length is 29 nt. All of the 75 nuclear scaffolds that were examined have at least one region that shows homology to ptDNA.

In total, 65.4 kb (~0.06%) of the *V. carteri *nuclear-genome-sequence data that were analyzed share sequence identity to the short palindromic repeats of the organelle genomes. The secondary structure and general characteristics of the nuclear-palindromic repeat elements are the same as those described for the organelle palindromes in Figures 5 and 6.

In order to place the *V. carteri *data described above in a broader context, we analyzed the *C. reinhardtii *nuclear genome for regions that show sequence identity to its mtDNA and ptDNA – these results are summarized in Supplementary Table S2 [see Additional file 5]. Only 0.007% of the *C. reinhardtii *nuclear genome maps to organelle DNA (0.0035% to the mtDNA and 0.0035% to the ptDNA), which is 10-times less than what is observed for *V. carteri *(0.01%).

## Discussion

### Expanded architectures and the proliferation of selfish DNA in the organelle genomes of *V. carteri*

The *V. carteri *organelle genomes are profuse with noncoding DNA. Our analyses indicate that the mitochondrial genome is >61% noncoding, which is the most expanded chlorophyte-mtDNA sequence currently deposited in Genbank [30] – other complete mitochondrial genome sequences from chlorophyte are larger only because they contain more genes. The plastid genome is the largest (>420 kb) and most bloated (>81% noncoding) ptDNA sequence from any taxon observed to date (Figure 4). Heretofore, the largest documented plastid genome was 223.9 kb, belonging to the chlorophyte *Stigeoclonium helveticum *[31]; and the plastid genome with the highest fraction of noncoding DNA was that of *C. reinhardtii *(56% noncoding) [6]. At present, due to a lack of available mtDNA and ptDNA sequence data, we do not know if other colonial or multicellular algae in the volvocine line of the Chlorophyceae have bloated organelle genomes as a result of excessive noncoding DNA. Preliminary investigations of the *Volvox aureus *mitochondrial genome indicate that it harbours palindromic repeats; these repeats do not share sequence similarity with those of the *V. carteri *mtDNA [17].

The fact that short palindromic repeats respectively constitute ~80% and ~75% of the mtDNA and ptDNA noncoding regions suggests that these elements precipitated the expansion of the organelle genomes. We must, therefore, ask: how did the palindromic repeats disseminate their sequence throughout the noncoding regions of the organelle DNA? The processes by which this could have occurred include transposition, retrotransposition via an RNA intermediate, and recombination-based mechanisms, such as gene conversion (for a review on the mobility of selfish DNA see Austin and Trivers [32]); at present, we are not partial to any one of these mechanisms. It is worth noting, however, that both the mitochondrial and plastid genomes of *V. carteri *encode a putative reverse transcriptase and a putative endonuclease; the mutual association of these enzymes has been invoked for mediating the retrotransposition of selfish DNA. Koll et al. [33] propose that the mobility of an ultra-short invasive element in the mtDNA of the filamentous fungus *Podospora anserina *is instigated by a group-II-intron-encoded reverse transcriptase; they also suggest that an intron-encoded endonuclease generates the 3'-hydroxyl required for reverse transcription. Of all the intronic ORFs deposited in Genbank, the deduced amino-acid sequence of *ORF261 *in the *Volvox *mtDNA shows the greatest identity (46%; expectation values = 6 × 10^-48^) to that of the *cox1*-intronic ORF of *P. anserina*. Another intriguing observation is that the short palindromic repeats in the *V. carteri *organelle and nuclear genomes are found in both orientations on the same strand, i.e., the same sequence can occur in the 3' to 5' and 5' to 3' directions – this is suggestive of a transposition-mediated mechanism of mobility rather than one based on recombination.

### Short palindromic repeats in the three genetic compartments of *V. carteri*

Short palindromic repeats are found in the three genetic compartments of *V. carteri*. The palindromic elements of the mtDNA are structurally similar but different in nucleotide sequence to those of the ptDNA, whereas the nucDNA palindromes are of two types: those that map to the mtDNA and those that map to ptDNA. These observations evoke several questions, such as are the palindromic repeats in the mtDNA and ptDNA related? And if so, in what genetic compartment did they first appear? Moreover, how did the nucDNA acquire palindromes that share sequence identity to those of the mitochondrial and plastid genomes, and why are they not as abundant as those of the organelle DNA? And finally, are the palindromic elements indigenous to *V. carteri *or are they the products of lateral gene transfer?

Although the mtDNA palindromic repeats differ in nucleotide sequence to the palindromes of the ptDNA, these two groups of repeats share enough structural features to suggest that they have descended from a common repetitive element and, thus, represent a single class of selfish DNA. For instance, in both the mitochondrial and plastid compartments the palindromic repeats reside in identical genomic landscapes (intergenic regions and non-ORF portions of introns) and they also inhabit these noncoding regions in similar abundances, representing ~80% of the noncoding nucleotides in both the mitochondrial and plastid genomes. Moreover, the mtDNA and ptDNA palindromes fold into hairpin structures where the loop portion is consistently 5'-TAAA-3' or 5'-ATTT-3'.

The same palindromic elements that have propagated in the *V. carteri *organelle DNA are also found in a small fraction (~0.06%) of the nucDNA. Although these nuclear-palindromic repeats can share up to 100% sequence identity with those of the mtDNA or ptDNA, many are degenerate with mismatches in the stem component of their hairpin structures. This observation, coupled with the relatively low abundance of these repeats in the nuclear genome, leads us to suggest that the nucDNA palindromes are inert, accumulating in the nucDNA through both mitochondrial-to-nucleus and plastid-to-nucleus DNA-transfer events – the movement of organelle DNA to nuclear genomes is well documented [34-37]. Further evidence to support this hypothesis is the fact that the proposed organelle-derived palindromes are present within the nuclear genome in the same proportions as other genetic regions from the mitochondrial and plastid genomes, such as coding regions (Table 1).

Two observations regarding the derivation of the short palindromic repeats in *V. carteri *are worth noting. First, the palindromic elements appear to have a strong affinity for the non-ORF regions of organelle introns, especially those in the mitochondrial genome. Because organelle introns are, themselves, a type of selfish element, which can migrate between organelle compartments, both within a species and between unrelated species ([38,39], and see [40] for a discussion on volvocalean ptDNA group-I introns), it is not unreasonable to surmise that the origin of the short palindromic repeats in *V. carteri *is linked to lateral intron transfer. The second observation is that the short palindromic repeats have propagated in only two of the nine introns in the *V. carteri *plastid genome: the group-I introns of *psaA *and *atpA*. Why have the other seven plastid introns (one group-I and six group-II introns) remained inviolate from palindromic elements? The two group-I plastid introns that contain short palindromic repeats belong to subgroup IB. Considering that the other group-I intron in the ptDNA (that of *chlL*), which is devoid of palindromes, is also of subgroup IB, it seems unlikely that the palindromic elements are favouring a certain class of intron. It is noteworthy that the *chlL *group-I intron lacks a LAGLIDADG homing endonuclease, whereas the group-I introns of *psaA *and *atpA*, as well as those of the mtDNA, harbour LAGLIDADG ORFs – perhaps, as suggested above, the mobility of the palindromic elements is dependent on intronic endonuclease proteins. Another possibility is that the plastid genome was seeded with short palindromic repeats after the inception of palindromic elements in the mtDNA and, therefore, the plastid palindromic repeats have not had sufficient time to spread to all of the intronic regions in the ptDNA; moreover, some of ptDNA introns may have arrived more recently in evolutionary time than those of *psaA *and *atpA*, and, thus, have not yet been seeded with repeats.

### Short palindromic repeats and the evolution of fragmented ribosomal-RNA-coding modules

When Aono et al. [17] first identified short palindromic repeats in the *V. carteri *mitochondrial genome they predicted that these repeats would be associated with the fragmentation of rRNA-transcripts. The mtDNA data presented here may support these predications. Our annotation of the *V. carteri *mitochondrial genome contains eight modules encoding the LSU rRNA, and their arrangement within the mtDNA is identical to that of *C. reinhardtii*, with the exception that the L8 module of *V. carteri *contains a large block of palindromic repeats, whereas that of *C. reinhardtii *harbours no repetitive mtDNA. At the present time we do not know if this insertion represents a fragmentation point within the L8 coding module that is removed from the primary transcript or if it is maintained as a variable region within an intact L8 transcript. We favour the former possibility because in the latter scenario the insertion is three-fold larger than any previously reported variable region identified in ribosomal RNA – based on the Comparative RNA Website [41].

## Conclusion

The goal of this study was to investigate the genomic breadth and the evolutionary implications of short palindromic repeats in the organelle and nuclear genomes of *V. carteri*. Our findings indicate that selfish DNA, in the form of palindromic elements, have proliferated in the *V. carteri *mtDNA and ptDNA; and although copies of this element exist in the nuclear compartment, we suggest that they are inert and arrived in the nuclear genome via rare organelle-to-nucleus DNA transfer events. We speculate that the palindromic repeats in *V. carteri *descended from a single invasive element, perhaps first seeded in *V. carteri *through the lateral gene transfer of a mitochondrial intron, eventually spreading to the ptDNA through mitochondrial-to-plastid DNA migration. Overall, the palindromic repeats appear to be involved with the expansion of the *V. carteri *organelle DNA and have potentially precipitated a gene fragmentation event in the mitochondrial genome.

## Methods

### Strain and DNA extractions

The sequence data generated in this study were obtained from the 72-52 dissociator mutant of *V. carteri *(UTEX 2908), which is derived from *V. carteri *strain HK10 (UTEX 885) [23,24]. Total genomic DNA was extracted using the DNeasy Plant Mini Kit (Qiagen, Germantown, MD, USA) following manufacturer's protocol.

### Amplification, cloning, and sequencing of DNA fragments

The organelle loci examined in this study were amplified using a PCR-based approach. PCR reactions were performed with the LongRange PCR Kit (Qiagen) using total genomic DNA as the template. PCR products were cloned using the TOPO TA Cloning Kit (Invitrogen, Carlsbad, CA, USA). Purified PCR products and isolated plasmids were sequenced on both strands at the Macrogen Sequencing Facility, Rockville, MD.

### Assembly of the mitochondrial and plastid DNA sequences

Sequences were edited and assembled using CodonCode Aligner Version 2.0.6 (CodonCode Corporation, Dedham, MA, USA), which employs the Phred, Cross-match, and Phrap algorithms for base calling, sequence comparison, and sequence assembly, respectively. Assemblies were performed with a minimum-percent-identity score of 98, a minimum-overlap length of 500 nt, a match score of 1, a mismatch penalty of -2, a gap penalty of -2, and an additional first gap penalty of -3.

### Sequence confirmation

The mtDNA and ptDNA data presented in this study were validated by collecting and assembling mitochondrial- and plastid-genome sequences that were generated by the *V. carteri *nuclear genome sequencing project [18]. These sequences were obtained by blasting our mtDNA and ptDNA data against the *V. carteri *f. *nagariensis *Whole Genome Shotgun Reads Trace Archive Database at the National Center for Biotechnological Information (NCBI) [42]. Blast hits showing >99% similarity to our *V. carteri *mtDNA and ptDNA sequences were downloaded and assembled (using the assembly program and parameters described above); the downloaded mitochondrial and plastid sequences were subsequently blasted against the *V. carteri *draft nuclear genome sequence (v1.0 Repeatmasked) [18] to verify that no nuclear-genome-located mtDNA-like or ptDNA-like sequences were collected.

### Analyses of repeats, introns, and intergenic regions

An initial scan for repetitive elements in the *V. carteri *mtDNA and ptDNA sequence data was performed with REPuter [43,44] using the Hamming distance option and a minimal-repeat-size setting of 12 nt – note, forward, reverse, complement, and reverse complement repeats were all considered under REPuter. Further analyses of the *V. carteri *organelle DNA repeats were performed in Geneious (Biomatters LtD, Auckland, NZ) by building a Blast databank of the mtDNA and ptDNA sequences and then blasting these databanks with specific regions from the mitochondrial and plastid genomes. Mfold [45] was employed for secondary-structure analyses. The mtDNA and ptDNA introns were detected, classified, and folded into secondary structures using RNAweasel [46,47]. The noncoding-DNA estimates presented for the *V. carteri *organelle genomes were inferred from the average-intergenic-spacer sizes and intron-densities of the mtDNA and ptDNA data that were collected. Dotplot similarity matrices were plotted with JDotter [48]. The LSU rRNA secondary-structure model depicted in Figure 3 is based on that of Boer and Gray [49]. The *C. reinhardtii *mtDNA-introns shown in Figure 1 are described elsewhere [50].

### Inspection of *V. carteri *nuclear DNA for organelle-like sequences

The *V. carteri *nucDNA was scanned for regions of identity to organelle DNA by blasting [51] the mtDNA and ptDNA sequences produced in this study against the *V. carteri *draft nuclear genome sequence (v1.0 Repeatmasked) [18] using an expectation value of 1 × 10^-5 ^and a word size of 11. Organelle-DNA sequences that mapped to the nuclear genome with >90% identity and at least 25 nt of aligned length were counted as hits. The same protocol employed for *V. carteri *was used to scan the *C. reinhardtii *nucDNA for regions that show identity to organelle sequences. The *C. reinhardtii *nucDNA scaffolds (version 3.1) were downloaded from the DOE JGI [52].

### Accession numbers

The mtDNA and ptDNA sequences generated in this study are found in Genbank under the accession numbers EU760701 and EU755264–EU755299, respectively.

## Authors' contributions

DRS carried out the molecular studies, data analyses, and wrote the manuscript. RWL helped in interpreting the data and revising the manuscript. Both DRS and RWL have read and approved the final version of this manuscript.

## Supplementary Material

Additional File 1 **Supplementary Table S1.** The fraction of noncoding DNA in completely-sequenced mitochondrial and plastid genomes from streptophytes, chlorophytes, and other plastid-harbouring taxa. Click here for file

Additional File 2 **Supplementary Figure S1.** Dotplot similarity matrix of the *Volvox carteri *mitochondrial DNA plotted against itself. Click here for file

Additional File 3 **Supplementary Figure S2.** Putative secondary-structure diagrams of the tRNA pseudogenes identified in the mitochondrial genome of *Volvox carteri*. Click here for file

Additional File 4 **Supplementary Figure S3.** Dotplot similarity matrix of the *Volvox carteri *plastid DNA plotted against itself. Click here for file

Additional File 5 **Supplementary Table S2.** Amount of nuclear DNA in *Chlamydomonas reinhardtii *that maps to its mitochondrial and plastid genomes. Click here for file
