# Supplementary material for: The mitochondrial and plastid genomes of Volvox carteri: bloated molecules rich in repetitive DNA
Source: BMC Genomics. 2009 Mar 26;10:132. doi: 10.1186/1471-2164-10-132 (PMC2670323; doi:10.1186/1471-2164-10-132)

Supplementary Figure S1 — Dotplot similarity matrix of the *Volvox carteri* mitochondrial DNA plotted against itself.

The X- and Y-axes each represent the *V. carteri* mtDNA sequence data (29,961 nt) that were obtained in this study; for clarity, a partial genetic map of the *V. carteri* mtDNA is placed below and beside these-axes, respectively (refer to Figure 1 for a full annotation of this genetic map). Dots in the nucleotide similarity matrix represent regions of shared identity between the two sequences; this matrix was generated using a window size of 25 and a stringency of 21.

Supplementary Figure S1

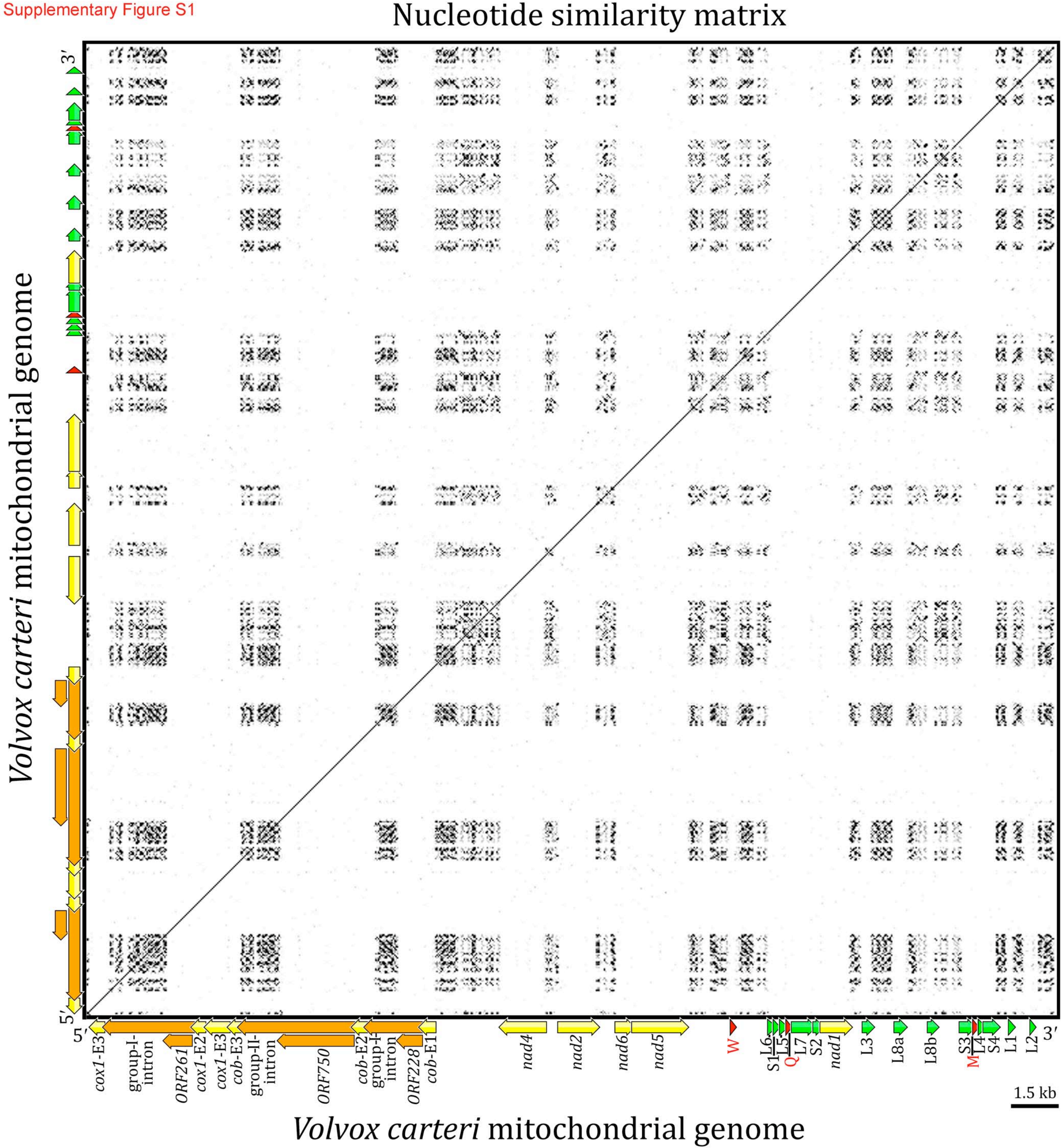

Supplement: Additional File 2 — Supplementary Figure S1. Dotplot similarity matrix of the Volvox carteri mitochondrial DNA plotted against itself. [file 1471-2164-10-132-S2.pdf]
