# Supplementary material for: The mitochondrial and plastid genomes of Volvox carteri: bloated molecules rich in repetitive DNA
Source: BMC Genomics. 2009 Mar 26;10:132. doi: 10.1186/1471-2164-10-132 (PMC2670323; doi:10.1186/1471-2164-10-132)

Supplementary Figure S2 — Putative secondary-structure diagrams of the tRNA pseudogenes identified in the mitochondrial genome of *Volvox carteri*.

Solid lines show Watson-Crick base pairings. Black dots denote G•T base pairings. The anticodon portions of the tRNA pseudogenes are shaded in gray and their associated amino acids are labeled below the cloverleaf structures. Insertions in the tRNA pseudogenes are signified with solid red arrows followed by the length (nt) of the insertion. Twelve copies and one copy of tRNA pseudo genes A and B are present, respectively, in the *V. carteri* mitochondrial genome.

Supplementary Figure S2

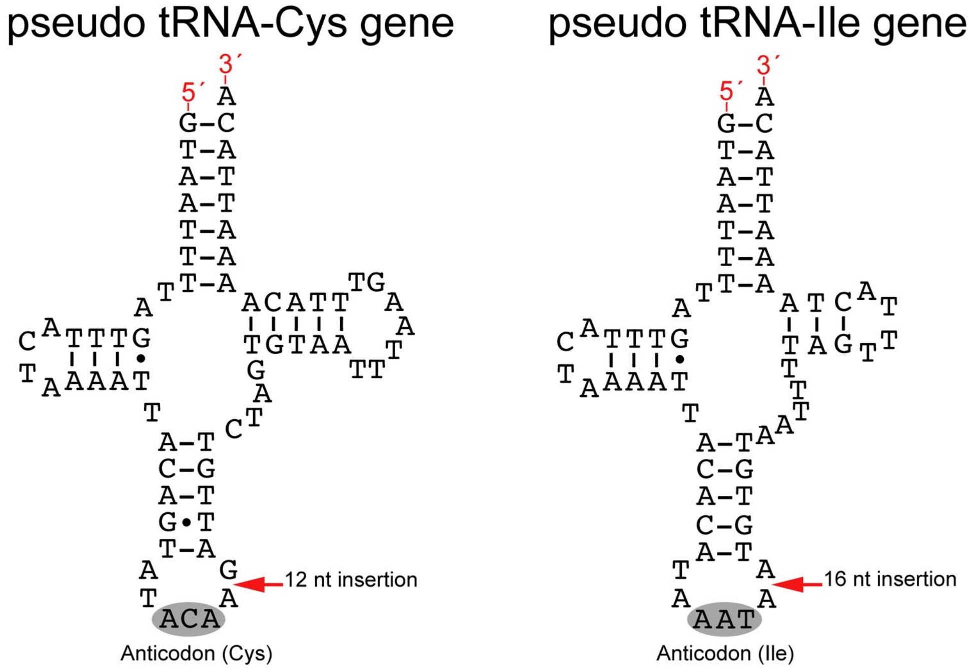

Supplement: Additional File 3 — Supplementary Figure S2. Putative secondary-structure diagrams of the tRNA pseudogenes identified in the mitochondrial genome of Volvox carteri. [file 1471-2164-10-132-S3.pdf]
