# Supplementary material for: The mitochondrial and plastid genomes of Volvox carteri: bloated molecules rich in repetitive DNA
Source: BMC Genomics. 2009 Mar 26;10:132. doi: 10.1186/1471-2164-10-132 (PMC2670323; doi:10.1186/1471-2164-10-132)

Supplementary Figure S3 — Dotplot similarity matrix of the *Vovox carteri* plastid DNA plotted against itself.

The X- and Y-axes each represent the *V. carteri* ptDNA sequence data (420,650 nt) that were obtained in this study — note: this sequence is a concatenation of the ptDNA contigs shown in Figure 2, starting with the *ropC1* contig and finishing with that of *petL*. For convenience, partial genetic map of the *V. carteri* ptDNA is placed below and beside the X and Y-axes, respectively; on this genetic map, all of the coding regions and intronic ORFs are shown in yellow. Dots in the nucleotide similarity matrix represent regions of shared identity between the two sequences; this matrix was generated using a window size of 25 and a stringency of 21.

Supplementary Figure S3

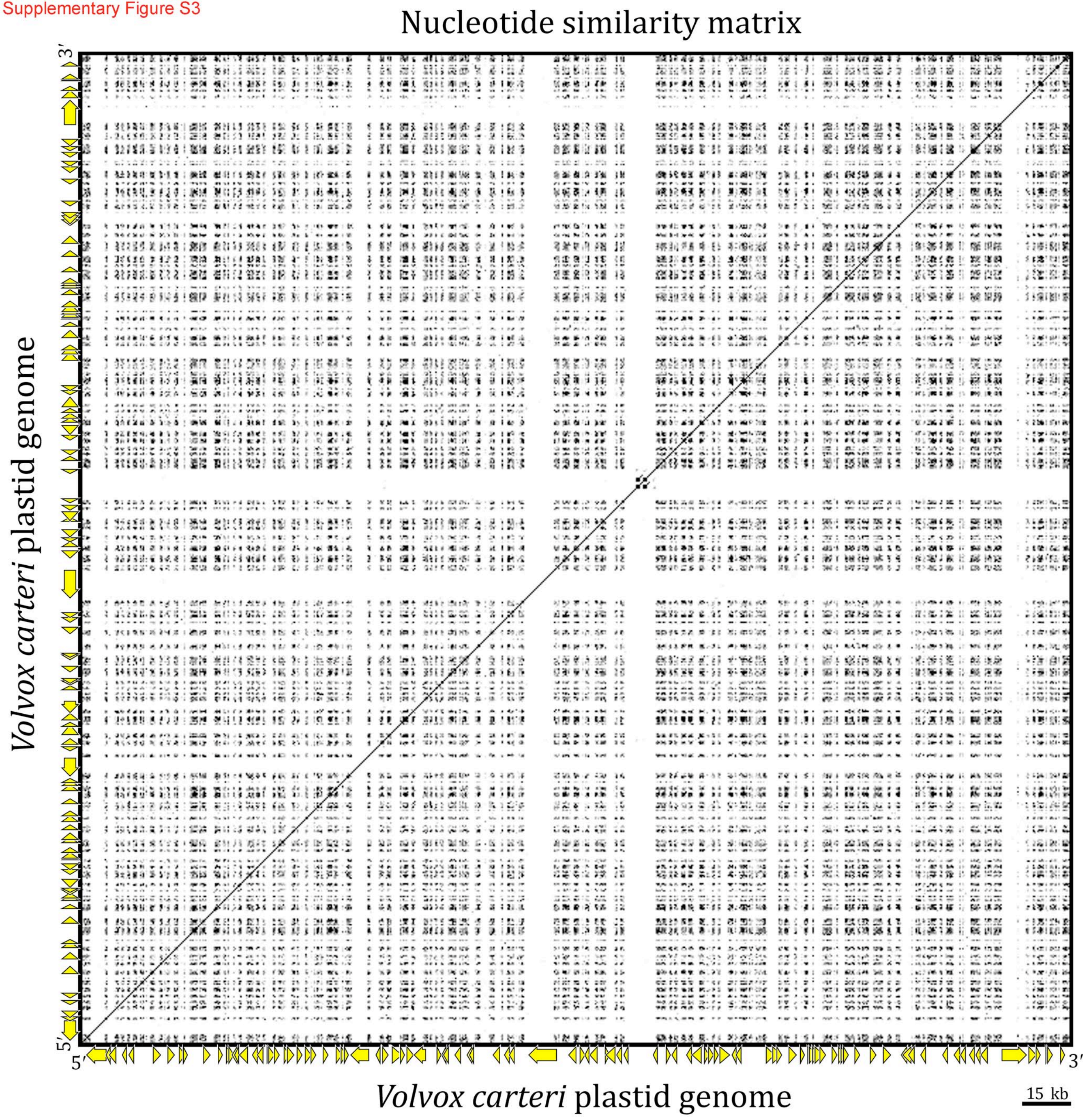

Supplement: Additional File 4 — Supplementary Figure S3. Dotplot similarity matrix of the Volvox carteri plastid DNA plotted against itself. [file 1471-2164-10-132-S4.pdf]
