# Supplementary material for: The mitochondrial and plastid genomes of Volvox carteri: bloated molecules rich in repetitive DNA
Source: BMC Genomics. 2009 Mar 26;10:132. doi: 10.1186/1471-2164-10-132 (PMC2670323; doi:10.1186/1471-2164-10-132)
Supplement: Additional File 5 — Supplementary Table S2. Amount of nuclear DNA in Chlamydomonas reinhardtii that maps to its mitochondrial and plastid genomes. [file 1471-2164-10-132-S5.pdf]

Supplementary Table S2 – Amount of nuclear DNA in *Chlamydomonas reinhardtii* that maps to the mitochondrial and plastid genomes.

		# of similarity regions[a]	Avg. length of similarity region (nt)	Max similarity length (nt)	Cumulative lengths of similarity regions (nt)	Fraction of nuclear genome
Amount of nucDNA mapping to the mitochondrial genome (by mtDNA subcategory[b])	Protein-coding genes[c]	34	61	333	2075	2.0×10^{-5}
	Structural-RNA genes[d]	7	40	57	281	0.3×10^{-5}
	Intronic ORFs[e]	13	32	43	413	0.4×10^{-5}
	Intergenic and non-ORF intronic regions[f]	16	50	206	751	0.7×10^{-5}
	Subtotal	70	55	333	3520	3.4×10^{-5}
Amount of nucDNA mapping to the plastid genome (by ptDNA subcategory[b])	Protein-coding genes[c]	34	44	129	1528	1.5×10^{-5}
	Structural-RNA genes[d]	12	50	94	596	0.6×10^{-5}
	Intronic ORFs[e]	---	---	---	---	---
	Intergenic and non-ORF intronic regions[f]	11	64	1093	1496	1.5×10^{-5}
	Subtotal	57	63	1093	3620	3.6×10^{-5}
Total amount of nucDNA that maps to organelle DNA		137	60	1093	7140	7.0×10^{-5}

Note: Nuclear DNA analyses are based on the *C. reinhardtii* draft nuclear genome sequence (version 3.1) at the DOE JGI [52]. Only the first 75 scaffolds of the nuclear-genome assembly were analyzed; approximately 84% of the *C. reinhardtii* nucDNA is contained in these 75 scaffolds and their cumulative length is 101.2 Mb.

[a] The number of distinct regions in the *C. reinhardtii* nucDNA that show >90% sequence identity and at least 25 nt of aligned length to organelle DNA.

[b] Refers to the region of the organelle genome to which the nucDNA maps.

[c] Includes all of the identified protein-coding genes.

[d] Includes all of the identified tRNA- and rRNA-coding genes.

[e] Includes all of the identified group I and II intronic-ORFs. Note, there are no intronic ORFs in the *C. reinhardtii* ptDNA.

[f] Includes all of the identified intergenic and non-ORF intronic regions.
